# Engineered mesenchymal stem cell-derived extracellular vesicles as programmable biomaterial platforms for liver fibrosis: design principles, manufacturing and clinical translation

**DOI:** 10.3389/fbioe.2026.1966076

**Published:** 2026-09-18

**Authors:** Fanghong Wang, Kexiang Zhu, Xiaoliang Zhu, Jia Yao, Zongbin Sun, Xun Li

**Affiliations:** 1 Department of General Surgery, The First Hospital of Lanzhou University, Lanzhou, Gansu, China; 2 The First School of Clinical Medicine, Lanzhou University, Lanzhou, Gansu, China; 3 Key Laboratory of Biotherapy and Regenerative Medicine of Gansu Province, Lanzhou, Gansu, China

**Keywords:** biomaterial platforms, engineering integration, mechanism-linked potency, nano-bio interface, quality-by-design, translational robustness

## Abstract

Liver fibrosis remains a major unmet clinical challenge characterized by persistent activation of hepatic stellate cells (HSCs), chronic inflammatory remodeling, and impaired hepatocyte regeneration. Although mesenchymal stem cell-derived extracellular vesicles (MSC-EVs) have emerged as promising cell-free therapeutics, native EVs are limited by insufficient target specificity, rapid systemic clearance, heterogeneous cargo composition, and poorly defined dose-response relationships, thereby constraining their translational potential. Recent advances in bioengineering have redefined MSC-EVs as programmable nanobiomaterial platforms rather than passive biological byproducts. Through multilevel engineering, including parent cell modification, cargo modulation, surface functionalization, and biomaterial-assisted delivery, MSC-EVs can be rationally designed to modulate key fibrogenic pathways, reprogram immune microenvironments, restore extracellular matrix homeostasis, and enhance hepatocyte regeneration. Concurrently, innovations in scalable three-dimensional bioprocessing, good manufacturing practice-compatible purification, and mechanism-linked potency assays are accelerating the transition of engineered EVs toward clinically viable products. This review integrates pathophysiological targeting with multilevel engineering strategies, with particular emphasis on nano-bio interface design, quality-by-design manufacturing, critical quality attribute definition, and key translational considerations, including dosing, safety, and regulatory frameworks. By positioning engineered MSC-EVs as precision biomaterial systems with controllable composition, programmable functionality, and tunable pharmacokinetics, we propose a rational design paradigm that bridges biological efficacy with scalable manufacturing and regulatory readiness. Collectively, such engineering integration is poised to transform EV-based antifibrotic therapy from experimental promise into clinically actionable intervention.

## 1 Introduction

Liver fibrosis represents a conserved wound-healing response to chronic liver injury and remains a principal driver of cirrhosis, portal hypertension, and liver-related mortality. Despite advances in etiological therapies, effective antifibrotic interventions capable of directly and consistently reversing established fibrosis remain lacking. At the mechanistic level, fibrosis progression is governed by the activation of hepatic stellate cells (HSCs), sustained inflammatory signaling, and dysregulated extracellular matrix (ECM) remodeling, ultimately accompanied by impaired hepatocyte regenerative capacity (Chiabotto et al., 2025; Yu S et al., 2025).

Mesenchymal stem cells (MSCs) have been extensively explored for liver diseases due to their immunomodulatory and trophic effects. However, the clinical translation of cell-based therapies remains constrained by multiple challenges, including donor- and culture-dependent heterogeneity, limited *in vivo* engraftment, potential safety risks such as microvascular embolism, and the lack of standardized definitions for product identity, dosing, and potency (Sitbon et al., 2024). Accumulating evidence indicates that a substantial proportion of MSC therapeutic efficacy is mediated through secreted extracellular vesicles (EVs), particularly small EVs commonly referred to as exosomes. MSC-derived EVs (MSC-EVs) function as nanoscale biological carriers capable of transferring nucleic acids, proteins, lipids, and metabolites to recipient cells, thereby modulating fibrogenic and inflammatory signaling pathways. Compared with live-cell therapies, MSC-EVs offer advantages including lower immunogenicity, improved biosafety profiles, and greater feasibility for storage and distribution (Kordelas et al., 2014; Yeo et al., 2013).

However, native MSC-EVs are not inherently optimized as therapeutic agents. They are typically limited by suboptimal target specificity, rapid systemic clearance, batch-to-batch variability, and incomplete definition of mechanism and potency. To address these limitations, engineering strategies have emerged as a central paradigm to enhance the therapeutic performance of MSC-EVs. These strategies operate at multiple levels, including parental MSCs priming or genetic modification, cargo loading and editing, surface functionalization, and integration with biomaterial-based delivery systems to improve targeting, biodistribution, and retention within fibrotic liver tissue (Lener et al., 2015; Toh et al., 2017). Representative approaches include preconditioning-driven cargo modulation, ligand-mediated surface engineering, and smart delivery platforms enabling spatiotemporally controlled release (Kooijmans et al., 2016). In parallel, the establishment of community guidelines such as MISEV2023 highlights the necessity for rigorous EVs characterization, standardized reporting, and functional validation to ensure reproducibility and facilitate clinical translation (Welsh et al., 2024; Witwer and Théry, 2019).

In this review, we systematically integrate the mechanistic foundations and engineering strategies underlying MSC-EV-based therapies for liver fibrosis. Particular emphasis is placed on rational delivery design, scalable manufacturing, quality control, and mechanism-linked potency assessment, as well as key translational considerations, including dosing strategies, safety evaluation, and regulatory frameworks (Gimona et al., 2021; Andaloussi et al., 2013). Within this framework, MSC-EVs are conceptually reframed not merely as biological mediators, but as biointerface-engineered nanomaterial systems with tunable composition, controllable pharmacokinetics, and programmable therapeutic functions. To provide a conceptual overview, Figure 1 integrates the key pathophysiological drivers of liver fibrosis with the major engineering modules used to reprogram MSC-EVs for antifibrotic therapy.

**FIGURE 1:**
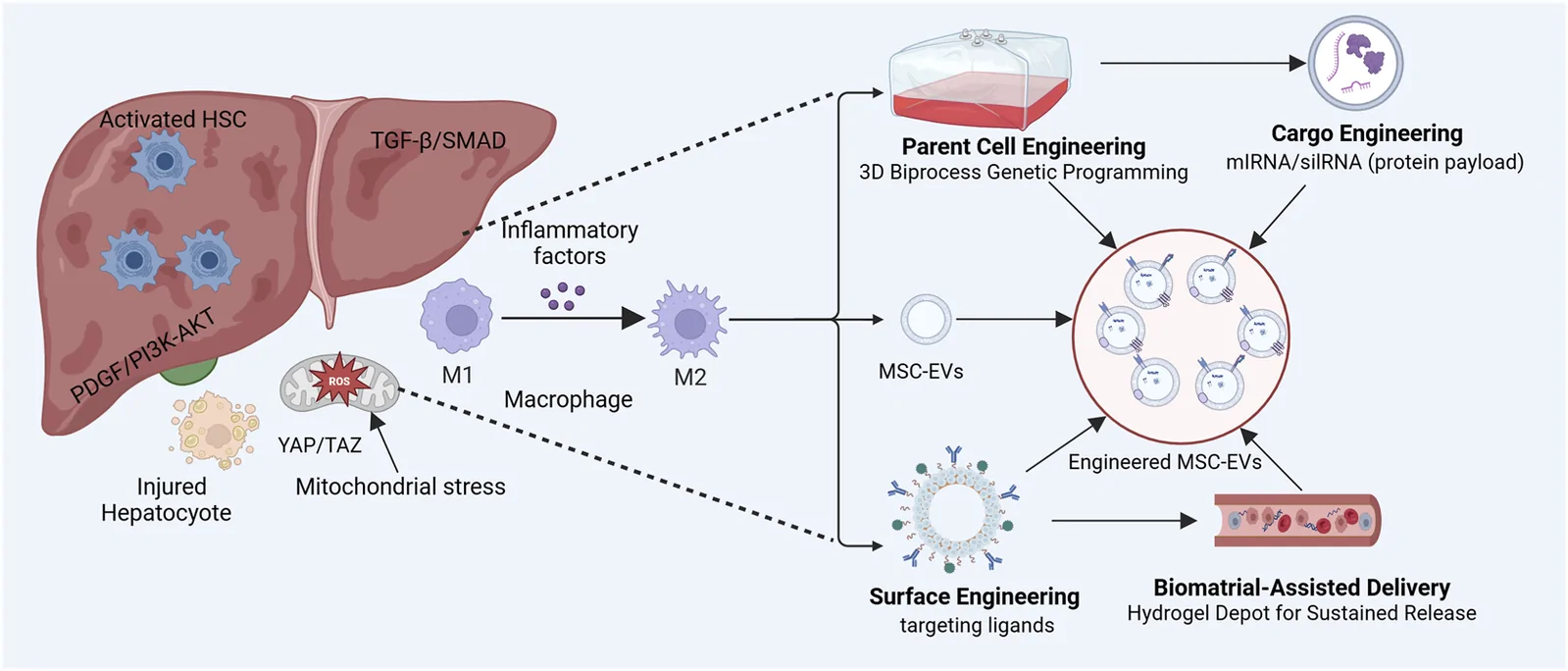
Conceptual framework of engineered MSC-EVs integrating disease mechanisms and multilevel engineering strategies for liver fibrosis. Liver fibrosis is driven by interconnected pathological processes, including HSC activation through TGF-β/SMAD, PDGF/PI3K–AKT, and YAP/TAZ signaling; macrophage-mediated inflammation; hepatocyte injury; mitochondrial stress; and ROS accumulation. Engineered MSC-EVs are designed to target these processes through four complementary strategies: parent-cell engineering using three-dimensional bioprocessing and genetic programming; cargo engineering with miRNAs, siRNAs, or therapeutic proteins; surface engineering with targeting ligands; and biomaterial-assisted delivery for sustained release. Together, these approaches enhance EV production, cargo potency, cellular targeting, and tissue retention, thereby enabling coordinated regulation of HSC activation, macrophage polarization, and hepatocyte injury. Abbreviations: MSC-EVs, mesenchymal stem cell-derived extracellular vesicles; HSC, hepatic stellate cell; TGF-β, transforming growth factor-β; SMAD, Sma- and Mad-related protein; PDGF, platelet-derived growth factor; PI3K, phosphoinositide 3-kinase; AKT, protein kinase B; YAP, Yes-associated protein; TAZ, transcriptional coactivator with PDZ-binding motif; ROS, reactive oxygen species; M1, classically activated macrophage phenotype; M2, alternatively activated macrophage phenotype; 3D, three-dimensional.

### 1.1 Literature search strategy

This narrative review was informed by searches of PubMed/MEDLINE, Web of Science Core Collection, and Scopus from database inception through August 2026. Search terms combined concepts related to mesenchymal stromal cells, extracellular vesicles or exosomes, liver or hepatic fibrosis, and EV engineering, including targeted delivery, cargo loading, surface modification, biomaterials, biodistribution, pharmacokinetics, safety, manufacturing, and quality control. Google Scholar and the reference lists of relevant articles were also examined to identify additional seminal or recent studies. Peer-reviewed original studies, relevant reviews, consensus statements, and methodological or regulatory guidance were considered. Because direct evidence on engineered MSC-derived extracellular vesicles (MSC-EVs) in liver fibrosis remains limited, mechanistically relevant studies involving other EV sources, fibrotic diseases, or delivery platforms were selectively included, with such extrapolations identified where appropriate. Conference abstracts, non-peer-reviewed reports, and studies unrelated to EV-based therapy or the scope of this review were excluded. Evidence was synthesized qualitatively and organized by mechanistic, engineering, pharmacological, manufacturing, and translational themes. Recent original and comparative studies were prioritized, while seminal studies were retained to support fundamental concepts. Given the heterogeneity of EV sources, engineering methods, experimental models, and outcome measures, no meta-analysis or formal risk-of-bias assessment was performed. This article is therefore presented as a narrative review rather than a systematic review or meta-analysis.

## 2 Pathophysiological targets in liver fibrosis relevant to EV-based therapy

Liver fibrosis is driven by persistent hepatic stellate cell activation, inflammatory crosstalk, and progressive extracellular matrix remodeling under the influence of mechanical and immunological signals. These processes operate within a highly interconnected cellular network involving hepatocytes, macrophages, endothelial cells, and stromal compartments. The dynamic interplay among these components establishes a self-reinforcing fibrogenic niche that ultimately progresses toward cirrhosis. The cellular, molecular, and mechanobiological landscape underlying fibrosis progression, together with its actionable therapeutic nodes, is summarized in Figure 2.

**FIGURE 2:**
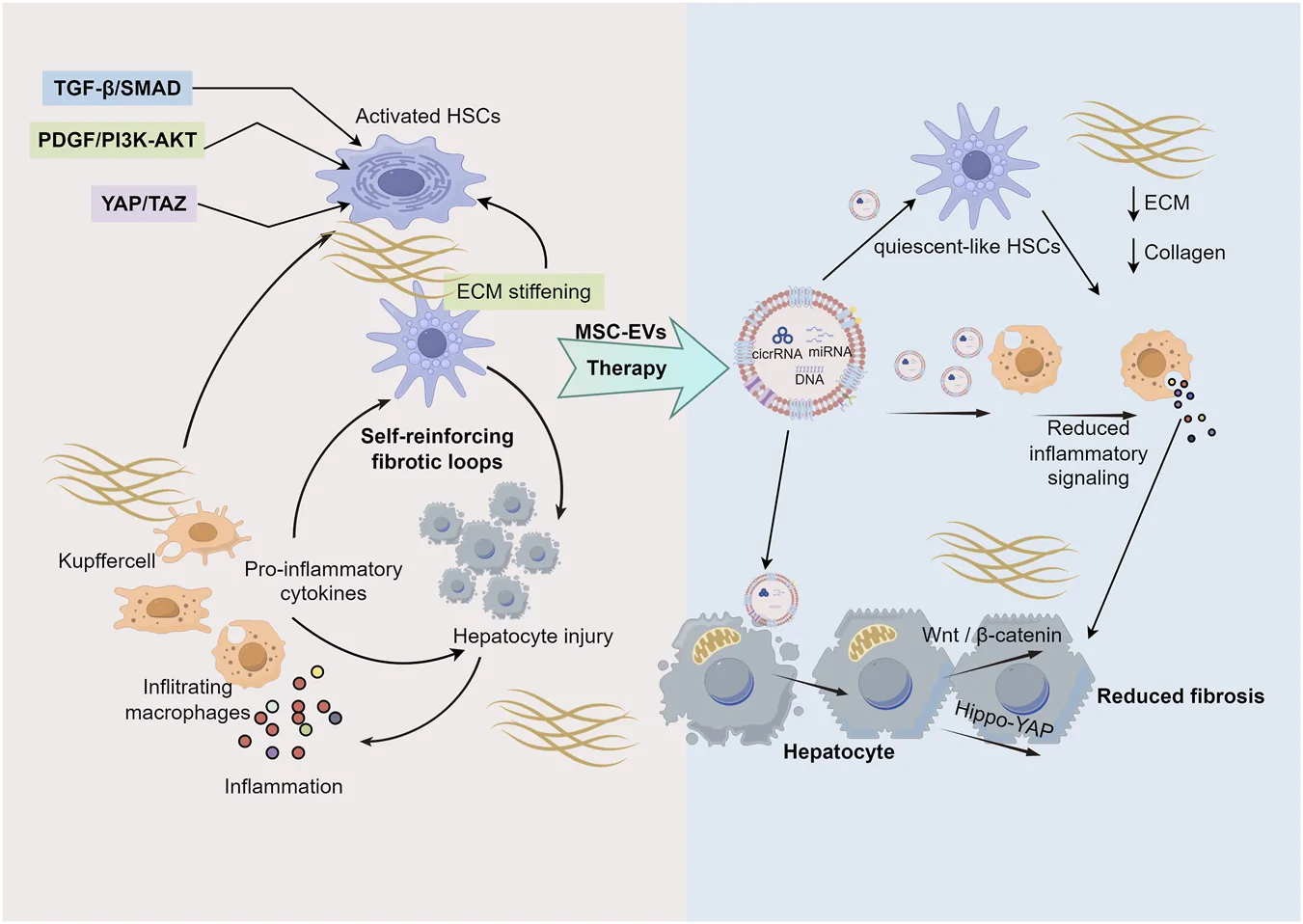
Pathophysiological network of liver fibrosis and actionable cellular targets for engineered MSC-EV therapy. Chronic hepatocyte injury activates Kupffer cells and recruits infiltrating macrophages, whose pro-inflammatory mediators amplify hepatocyte damage and HSC activation. TGF-β/SMAD and PDGF/PI3K–AKT signaling, together with ECM stiffness-induced YAP/TAZ mechanotransduction, sustains HSC activation and matrix deposition, thereby establishing self-reinforcing fibrotic loops. Engineered MSC-EVs deliver bioactive cargos, including circRNAs, miRNAs, and DNA, to key hepatic cell populations. These vesicles promote the reversion of activated HSCs toward a quiescent-like state, reduce ECM and collagen deposition, suppress macrophage-mediated inflammatory signaling, and enhance hepatocyte repair through modulation of Wnt/β-catenin and Hippo–YAP signaling. Together, these effects disrupt the fibrotic network and promote fibrosis resolution. Abbreviations: MSC-EVs, mesenchymal stem cell-derived extracellular vesicles; HSCs, hepatic stellate cells; TGF-β, transforming growth factor-β; SMAD, Sma- and Mad-related protein; PDGF, platelet-derived growth factor; PI3K, phosphoinositide 3-kinase; AKT, protein kinase B; ECM, extracellular matrix; YAP, Yes-associated protein; TAZ, transcriptional coactivator with PDZ-binding motif; Wnt, Wingless-related integration site.

### 2.1 HSCs activation and ECM remodeling

Activated hepatic stellate cells (HSCs) are the principal effector cells driving fibrogenesis and myofibroblast accumulation in chronic liver disease. Upon liver injury, quiescent, vitamin A-storing HSCs transdifferentiate into a proliferative, contractile, and ECM-producing phenotype. This activation process is orchestrated by complex paracrine and autocrine cues, including transforming growth factor-β (TGF-β), platelet-derived growth factor (PDGF), and mechanotransduction signals that arise from the stiffened ECM microenvironment (Higashi et al., 2017; Wells, 2008; Wynn and Ramalingam, 2012). Transforming growth factor-β (TGF-β)/SMAD signaling constitutes the canonical axis driving HSCs activation, inducing the transcription of profibrotic genes, including collagen type I and III, α-smooth muscle actin (α-SMA), and tissue inhibitor of metalloproteinase-1 (TIMP-1)(Dooley & ten Dijke, 2012). In parallel, platelet-derived growth factor (PDGF)-BB and PDGF-C function as potent mitogens that stimulate HSCs proliferation and migration predominantly through activation of the PI3K–Akt and ERK signaling pathways (Borkham-Kamphorst and Weiskirchen, 2016; Troeger et al., 2012). Concurrently, progressive ECM stiffening reinforces HSCs activation via integrin-mediated mechanotransduction, Yes-associated protein (YAP)/transcriptional coactivator with PDZ-binding motif (TAZ) signaling, and RhoA-ROCK2 activation, thereby establishing a self-amplifying positive feedback loop that sustains fibrogenesis (Dupont et al., 2011; Olsen et al., 2011). Activated HSCs secrete excessive ECM components, including collagen I, collagen III, and fibronectin, while disrupting the dynamic balance between matrix metalloproteinases (MMPs) and their tissue inhibitors (TIMPs). Dysregulation of MMP-2, MMP-9, TIMP-1, and TIMP-2 results in impaired ECM degradation and excessive matrix accumulation, ultimately promoting tissue stiffening and compromising hepatocyte regenerative capacity (Iredale, 2007; Nagase and Brew, 2003). Emerging therapeutic strategies therefore focus on three principal axes: (i) inhibition of upstream profibrotic signaling pathways, such as transforming growth factor-β receptor (TGF-βR) and platelet-derived growth factor receptor (PDGFR); (ii) induction of hepatic stellate cells apoptosis or senescence to limit myofibroblast accumulation; and (iii) restoration of ECM homeostasis through modulation of the MMP/TIMP axis. Beyond molecular targeting, biomaterial-based strategies have emerged as promising approaches to modulate the fibrotic microenvironment. In particular, matrix-degradable scaffolds and stiffness-tunable hydrogels are increasingly engineered to regulate HSCs mechanosensing, thereby enabling the reprogramming of fibrotic niches toward a regenerative phenotype (Caliari and Burdick, 2016; Chaudhuri et al., 2020).

### 2.2 Immune dysregulation and the inflammatory niche

The fibrotic liver establishes and sustains a chronic inflammatory microenvironment that critically governs both fibrogenesis and its potential resolution. Resident Kupffer cells and infiltrating monocyte-derived macrophages constitute the central immune compartment of this niche, acting in concert with neutrophils, natural killer (NK) cells, and lymphocyte subsets to coordinate tissue remodeling and injury repair (Ramachandran et al., 2012; Tacke and Zimmermann, 2014). Persistent liver injury activates pattern-recognition receptor signaling and drives the release of pro-inflammatory cytokines (e.g., TNF-α, IL-1β, and IL-6) and chemokines (e.g., CCL2 and CXCL10), thereby exacerbating hepatocyte stress while reinforcing HSCs activation and ECM deposition (Heymann and Tacke, 2016; Koyama and Brenner, 2017; Seki and Schwabe, 2015). During the early phase of injury, Kupffer cells predominantly exhibit a pro-inflammatory (M1-like) phenotype, characterized by the production of nitric oxide and reactive oxygen species that propagate necroinflammation. In contrast, under pro-resolving conditions, Kupffer cells or recruited monocyte-derived macrophages can undergo phenotypic reprogramming toward M2-like states, secreting anti-inflammatory mediators such as IL-10 and TGF-β3, along with matrix-degrading enzymes including MMP-9, thereby facilitating fibrosis resolution (Duffield et al., 2005; Murray and Wynn, 2011; Ramachandran et al., 2012; Wynn and Vannella, 2016).

Recent advances in single-cell RNA sequencing and spatial transcriptomics have delineated discrete macrophage subpopulations within fibrotic livers, particularly localized to fibrotic septa and perivascular niches. These subsets are characterized by distinct transcriptional signatures, including elevated expression of SPP1, TREM2, and MARCO, highlighting the functional heterogeneity and spatial zonation of macrophage activity (Li et al., 2023; Ramachandran et al., 2019). These findings underscore the bidirectional plasticity of macrophages, which can either sustain chronic inflammation or actively promote matrix remodeling and hepatocyte regeneration, depending on microenvironmental cues. Accordingly, immunomodulation has evolved from a supportive strategy to a central paradigm in antifibrotic therapy design. Therapeutic approaches targeting macrophage biology—including inhibition of monocyte recruitment (e.g., CCR2/CCR5 antagonism), polarization toward pro-resolving phenotypes, and macrophage-based or EV-mediated immunotherapies—have demonstrated considerable potential to attenuate inflammation and enhance tissue repair (Bansal, Nagórniewicz and Prakash, 2016; Friedman et al., 2018). Collectively, these insights position the orchestration of the immune landscape, particularly the macrophage niche, as a key determinant of fibrosis regression rather than a secondary or adjunctive mechanism.

### 2.3 Hepatocyte injury and impaired regeneration

Persistent hepatocellular stress driven by metabolic overload, oxidative injury, and mitochondrial dysfunction serves as a central initiating factor in chronic inflammation and subsequent fibrogenesis. Hepatocyte apoptosis and necroptosis result in the release of damage-associated molecular patterns (DAMPs), which activate Kupffer cells and infiltrating macrophages, thereby amplifying inflammatory cascades and promoting ECM deposition (Krenkel and Tacke, 2017; Schwabe and Luedde, 2018; Tsuchida and Friedman, 2017). In parallel, impaired mitochondrial bioenergetics and enhanced lipid peroxidation compromise hepatocyte viability and regenerative capacity, further reinforcing the fibrotic cycle (Zhao et al., 2023). Regenerative failure in chronic liver disease arises from disrupted crosstalk between proliferative and inhibitory signaling pathways. Under physiological conditions, hepatocyte regeneration is tightly regulated by the Wnt/β-catenin and Hippo-YAP/TAZ signaling axes, which integrate mechanical and metabolic inputs to control cell proliferation, differentiation, and organ size (Monga, 2015; Yimlamai et al., 2014). During fibrosis, persistent mechanical stress and sustained TGF-β signaling impair regenerative competence by restricting YAP/TAZ activity and attenuating β-catenin signaling (Mannaerts et al., 2015). From a therapeutic perspective, restoring hepatocyte function requires not only suppression of injurious stimuli but also reactivation of regenerative signaling networks. In this context, EVs have emerged as potent hepatoprotective mediators, capable of improving mitochondrial function, alleviating oxidative stress, and promoting hepatocyte proliferation through modulation of Wnt-YAP signaling crosstalk (Li et al., 2013; Lou et al., 2017a; Monga, 2015; Rong et al., 2019). Engineered MSC-EVs can serve as multifunctional delivery platforms, transporting antioxidant enzymes, mitochondrial regulatory factors, and regenerative microRNAs (e.g., miR-125b, miR-199a, and miR-122), thereby reprogramming hepatocyte survival pathways and promoting coordinated tissue repair (Tan et al., 2014; Wang et al., 2025).

Collectively, these interconnected pathophysiological modules define actionable therapeutic axes for EV-based liver interventions: (i) reprogramming of HSC-driven fibrogenic signaling, (ii) remodeling of the immune niche toward a pro-resolving phenotype, and (iii) enhancement of hepatocyte survival and regeneration through mitochondrial restoration and activation of proliferative signaling pathways.

## 3 Biological basis of MSC-EVs in liver fibrosis

Engineered MSC-EVs exert coordinated antifibrotic and pro-regenerative effects through multi-targeted modulation of key cellular compartments, including activated HSCs, macrophage polarization states, and hepatocyte function, while simultaneously restoring ECM homeostasis. By integrating the regulation of inflammatory signaling, profibrotic transcriptional programs, and mitochondrial integrity, these vesicles reprogram the fibrotic microenvironment toward a pro-resolving state. The integrated cellular and molecular mechanisms underlying these therapeutic effects are summarized in Figure 3.

**FIGURE 3:**
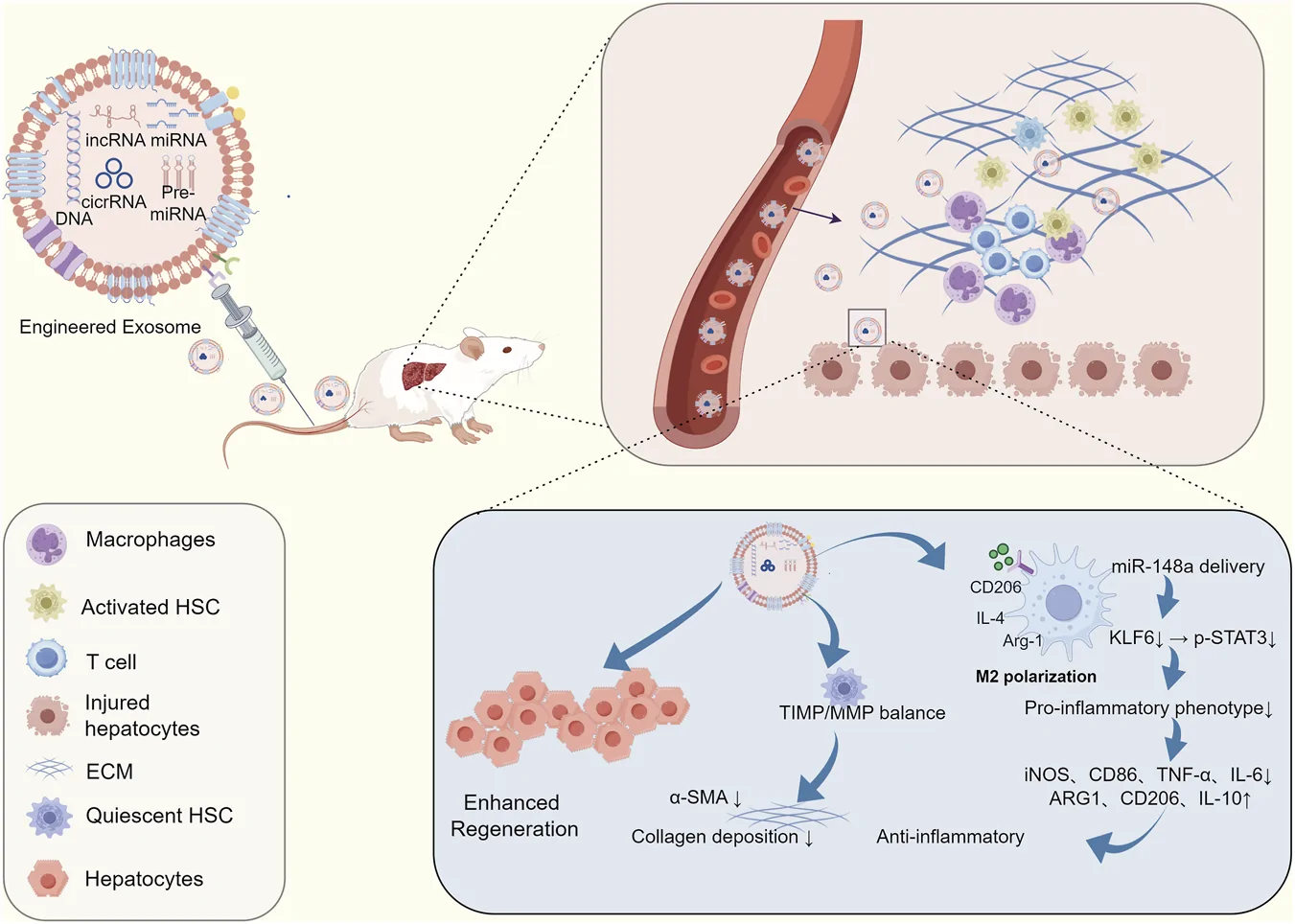
Therapeutic mechanisms of engineered MSC-EVs in liver fibrosis. Engineered MSC-EVs exert coordinated immunomodulatory, antifibrotic, matrix-remodeling, and regenerative effects. Following uptake by hepatic macrophages, EV-delivered miR-148a suppresses KLF6 and downstream STAT3 activation, promoting macrophage reprogramming toward an anti-inflammatory, M2-like/pro-resolving phenotype, characterized by increased Arg-1 and CD206 expression and decreased iNOS, TNF-α, IL-6, and CD86 expression. IL-4 is depicted as a canonical stimulus of M2-like macrophage polarization. In parallel, MSC-EVs attenuate HSC activation, restore MMP/TIMP homeostasis, reduce α-SMA expression and collagen deposition, and promote hepatic regeneration, collectively facilitating fibrosis resolution. Abbreviations: MSC-EVs, mesenchymal stem cell-derived extracellular vesicles; miR-148a, microRNA-148a; KLF6, Krüppel-like factor 6; STAT3, signal transducer and activator of transcription 3; M2, alternatively activated macrophage phenotype; Arg-1, arginase 1; CD206, cluster of differentiation 206; iNOS, inducible nitric oxide synthase; TNF-α, tumor necrosis factor-α; IL-4, interleukin-4; IL-6, interleukin-6; CD86, cluster of differentiation 86; HSC, hepatic stellate cell; MMP, matrix metalloproteinase; TIMP, tissue inhibitor of metalloproteinase; α-SMA, α-smooth muscle actin.

### 3.1 EVs cargo and intercellular communication

MSC-derived EVs serve as key mediators of intercellular communication by delivering diverse bioactive cargo, including microRNAs (miRNAs), long non-coding RNAs (lncRNAs), messenger RNAs, proteins, and lipids, to recipient cells, thereby modulating cellular function and signaling pathways. These molecular constituents collectively regulate gene transcription, epigenetic programs, and signal transduction across multiple hepatic cell types, including HSCs, Kupffer cells, macrophages, and hepatocytes (Valadi et al., 2007; Yu S. et al., 2025). The functional efficacy of MSC-EVs is highly context-dependent and is shaped by the cellular origin of MSCs (e.g., bone marrow, adipose tissue, umbilical cord, or menstrual blood), microenvironmental conditioning (such as hypoxia or cytokine priming), and downstream isolation, purification, and storage processes (Kalluri and LeBleu, 2020; Lötvall et al., 2014; Phinney and Pittenger, 2017). For example, EVs derived from hypoxia-preconditioned MSCs are enriched in miR-21 and hypoxia-inducible factor-1α (HIF-1α)-regulated proteins, which enhance hepatocyte survival and angiogenic responses (Hu et al., 2020).

In addition, engineered MSC-EVs carrying specific regulatory RNAs such as lncRNA H19 or miR-122 have demonstrated enhanced antifibrotic efficacy and improved capacity for metabolic reprogramming in liver disease models (Chen et al., 2023). Recent multi-omics analyses further reveal that lncRNA-miRNA-mRNA competing endogenous RNA (ceRNA) networks within MSC-EVs dynamically orchestrate key fibrogenic signaling pathways, including TGF-β/SMAD, NF-κB, and PI3K-Akt cascades, thereby reshaping the hepatic fibrotic niche (Chen et al., 2012; Statello et al., 2021).

Accordingly, precise characterization of EVs cargo composition, stability, and delivery kinetics is essential for elucidating therapeutic mechanisms and enabling robust clinical translation.

### 3.2 Reported therapeutic effects

Across diverse preclinical models of liver fibrosis—including carbon tetrachloride (CCl_4_) administration, bile duct ligation (BDL), and methionine–choline-deficient (MCD) diet-induced injury—MSC-EVs have consistently demonstrated antifibrotic efficacy, as evidenced by reduced α-SMA and collagen I expression, together with attenuation of hepatocyte apoptosis (Haga et al., 2017; Yan et al., 2017; Yuan et al., 2023; Zheng et al., 2023). Mechanistically, EV-delivered microRNAs, such as miR-122, miR-146a-5p, and miR-29a, directly suppress key HSC activation-associated genes, including COL1A1, ACTA2, and TIMP1, while modulating pro-inflammatory mediators such as IL-1β and CCL2 (Roderburg et al., 2011; Zhou et al., 2022). Furthermore, engineered MSC-EVs enriched in regulatory miRNAs, including miR-122 and miR-181–5p, exhibit enhanced antifibrotic potency by downregulating transforming growth factor-β receptor I (TGF-βRI) and suppressing SMAD2/3 phosphorylation in HSCs (Lou et al., 2017b).

Beyond these direct effects on HSCs, hepatic macrophages constitute a major immunological target of MSC-EVs within the fibrotic liver. Following internalization by resident Kupffer cells and recruited monocyte-derived macrophages, MSC-EV cargo can attenuate pro-inflammatory signaling and promote pro-resolving functional programs. For example, miR-148a-enriched MSC-EVs directly target KLF6 and attenuate downstream STAT3 activation, resulting in reduced expression of iNOS, TNF-α, IL-6, and CD86, together with increased expression of arginase-1, CD206, and IL-10 [64]. Enrichment of miR-148a further enhances the antifibrotic activity of MSC-EVs, illustrating how cargo engineering may strengthen immunomodulatory potency (Wiklander et al., 2015).

Parental-cell preconditioning provides another engineering strategy: small EVs derived from IFN-γ-conditioned MSCs are enriched in macrophage-regulatory proteins, including annexin A1, lactotransferrin, and aminopeptidase N, and more effectively induce anti-inflammatory macrophages with enhanced motility and phagocytic activity (Lai et al., 2013). At the tissue level, these responses may facilitate efferocytosis and matrix degradation, attenuate inflammatory macrophage–HSC crosstalk, and thereby promote fibrosis resolution. Nevertheless, macrophage activation occurs along a dynamic and context-dependent continuum rather than conforming to a strict M1/M2 dichotomy, and macrophage subsets may exert either profibrotic or fibrosis-resolving functions depending on their origin, activation state, and disease stage (Tacke and Zimmermann, 2014). Therefore, the immunomodulatory potency of engineered MSC-EVs should be evaluated using integrated phenotypic, cytokine, metabolic, transcriptomic, and functional readouts rather than relying exclusively on a limited set of M1/M2 markers.

### 3.3 Limitations of native MSC-EVs as therapeutics

Despite their considerable therapeutic promise, native MSC-EVs remain suboptimal as standalone interventions for liver fibrosis. Their translational limitations arise from several interconnected factors. First, unmodified EVs exhibit limited target specificity and delivery efficiency, with passive biodistribution largely governed by uptake in the liver and spleen via the mononuclear phagocyte system (MPS). Second, rapid systemic clearance results in short circulation half-lives and insufficient accumulation within fibrotic lesions. Third, poorly defined dose-exposure-response relationships complicate potency assessment and hinder regulatory evaluation. Fourth, substantial batch-to-batch variability arising from differences in MSC donor sources, culture conditions, and isolation or purification methods compromises reproducibility and product consistency (Gimona et al., 2017; Lai et al., 2013). In addition, the intrinsic molecular heterogeneity of EVs complicates product identity assignment and mechanistic attribution, posing further challenges for clinical standardization. In response, the updated MISEV2023 guidelines emphasize rigorous definition of EVs origin, marker profiling, and functional potency assays, advocating the integration of standardized characterization and validation pipelines (Witwer and Théry, 2019).

Collectively, these challenges highlight that engineering strategies are not merely advantageous but essential for advancing MSC-EVs from a biologically intriguing entity to a reproducible, scalable, and regulatory-compliant therapeutic platform.

## 4 Engineering strategies to enhance the therapeutic efficacy of MSC-Vs in liver fibrosis

Despite their intrinsic therapeutic potential, native EVs are constrained by limited targeting specificity, heterogeneous payload composition, and suboptimal pharmacokinetic profiles. To overcome these barriers, multilevel engineering strategies have been developed, encompassing parent-cell modification, cargo loading optimization, surface functionalization, and biomaterial-assisted delivery systems (Herrmann et al., 2021; Kojima et al., 2018; Kooijmans et al., 2012; Vader et al., 2016). Collectively, these approaches transform EVs from biologically derived nanoparticles into programmable therapeutic platforms with tunable functionality. A hierarchical overview of these engineering strategies, spanning source modification, cargo design, surface functionalization, and delivery systems, is presented in Table 1. To further enhance translational relevance, representative liver-fibrosis studies are comparatively summarized in Table 2, with emphasis on molecular design, biological target, therapeutic outcome, and major translational constraints, while their integrated functional mechanisms are illustrated in Figure 4.

**TABLE 1:** Engineering strategies for MSC-EVs therapy in liver fibrosis.

Engineering level	Strategy	Engineering advantage	Key challenges	Translational relevance
Parent UC-MSC	Genetic modification	Stable endogenous cargo enrichment	Regulatory complexity	High
Parent UC-MSC	Priming/preconditioning	Simple, non-genetic	Variability	Medium
Bioprocess	3D culture/bioreactor	Scalable EV yield	Process optimization	High
EV cargo	Endogenous loading	Potency consistency	Target selection	High
EV cargo	Exogenous loading	Flexible payload	EV integrity	Medium
EV surface	Ligand targeting	Enhanced liver uptake	Immunogenicity	Medium–High
Delivery system	Hydrogel/controlled release	Sustained exposure	Material safety	High

**TABLE 2:** Representative engineering strategies of MSC-EVs for liver fibrosis: molecular design, biological target, therapeutic outcome, and translational limitation.

MSC source	Engineering strategy	Cargo/ligand	Target cell	Key therapeutic outcome	Translational limitation	References
Adipose MSCs	Inflammatory priming (IFN-γ)	Protein-enriched EVs	Macrophages	Enhanced anti-inflammatory remodeling and fibrosis resolution	Batch variability and undefined active components	Liu et al. (2025)
Adipose MSCs	Genetic modification	miR-122	HSCs	Suppressed HSC activation and ECM deposition	Lack of targeting specificity and PK characterization	Kordelas et al. (2014)
Adipose MSCs	Genetic modification	miR-181–5p	HSCs	Inhibited fibrogenesis via autophagy activation	Limited translational validation and dosing standardization	Lőrincz Á et al. (2014)
Wharton’s jelly MSCs	Cargo enrichment	miR-124–3p	HSCs/monocytes	Reduced fibrosis and promoted immune resolution suppression	Complex immune effects complicate potency evaluation	Lötvall et al. (2014)
Umbilical cord MSCs	Surface engineering	aHSC-targeting peptide	HSCs	Enhanced cellular uptake and antifibrotic efficacy	Ligand density control and immunogenicity concerns	Lou et al. (2017a)
Stem cell-derived EVs	Surface engineering	Vitamin a ligand	HSCs	Improved targeting efficiency and reduced effective dose	Conjugation reproducibility and off-target distribution	Lou et al. (2017b)
MSCs	Cargo-defined strategy	miR-148a	Macrophages	Reprogrammed macrophage polarization and reduced fibrosis	Indirect mechanism and broad target effects	Lu et al. (2019)
Umbilical cord MSCs	Protein cargo enrichment	BECN1	HSCs	Induced ferroptosis of HSCs and decreased collagen deposition	Safety concerns related to oxidative stress	Lv et al. (2022)
Adipose MSCs	Combined engineering	MiR-150 + targeting peptide	HSCs	Synergistic suppression of fibrogenesis and improved liver function	Increased manufacturing complexity and regulatory burden	Mandenius and Brundin (2008)

This table summarizes representative MSC-EVs engineering strategies for liver fibrosis, highlighting that current approaches primarily enhance therapeutic efficacy through three major mechanisms: (i) direct suppression of hepatic stellate cell activation, (ii) modulation of macrophage polarization and inflammatory resolution, and (iii) improvement of targeting efficiency and pharmacokinetic profiles. Despite promising preclinical outcomes, translational challenges remain, particularly in standardization, large-scale manufacturing, and regulatory compliance.

**FIGURE 4:**
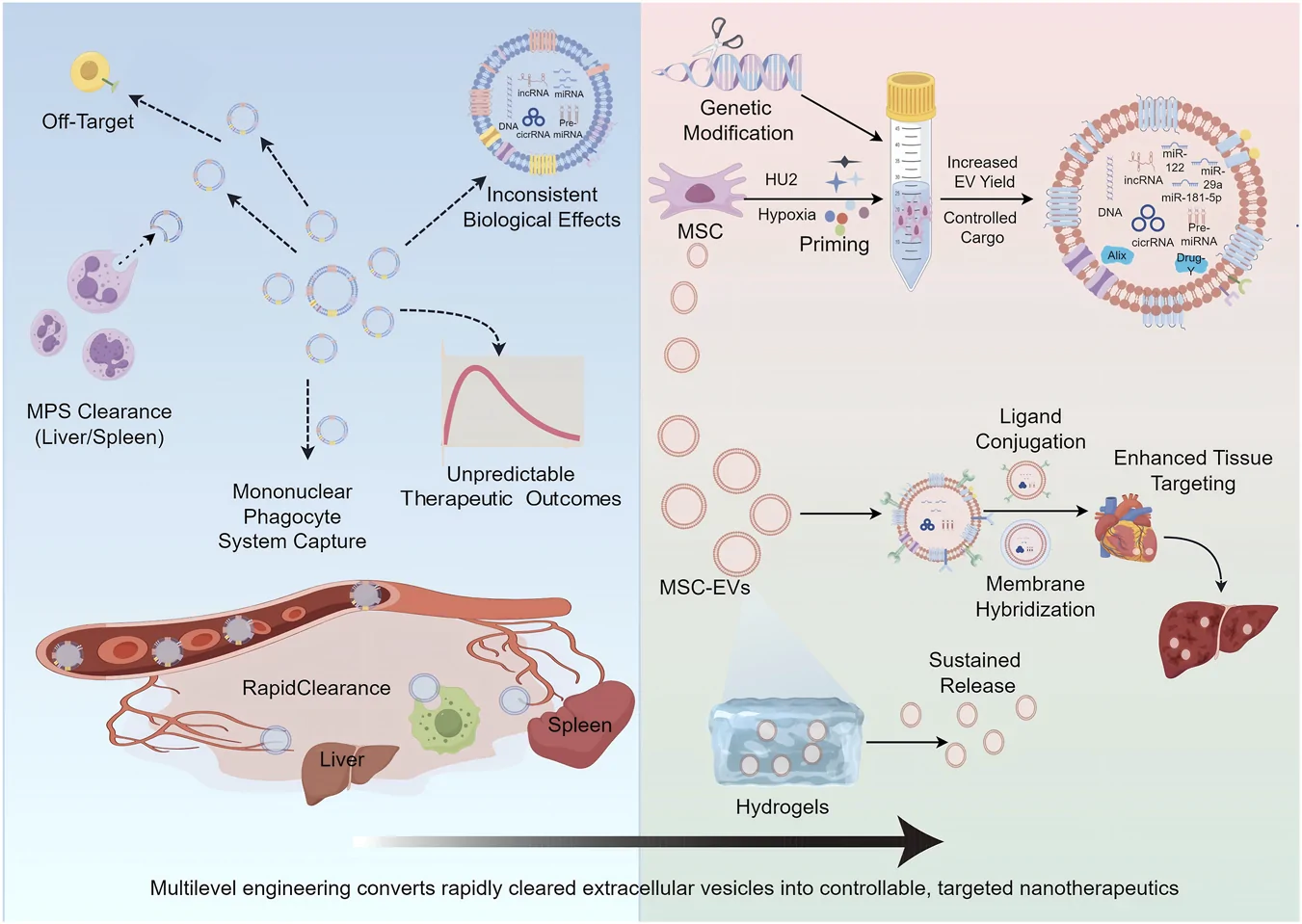
Limitations of native MSC-EVs and multilevel engineering strategies for therapeutic optimization. Native MSC-EVs are limited by rapid MPS-mediated capture and clearance, predominant sequestration in the liver and spleen, off-target distribution, heterogeneous cargo and biological activity, and consequently unpredictable therapeutic outcomes. Multilevel optimization includes genetic modification and priming of parental MSCs to increase EV yield and standardize cargo; loading of therapeutic nucleic acids, proteins, or drugs; ligand conjugation and membrane hybridization to enhance tissue targeting; and hydrogel-assisted delivery for sustained release. Collectively, these strategies transform native MSC-EVs into more controllable, targeted, and durable nanotherapeutic systems. Abbreviations: MSC, mesenchymal stem cell; EV, extracellular vesicle; MSC-EVs, mesenchymal stem cell-derived extracellular vesicles; MPS, mononuclear phagocyte system; ALIX, apoptosis-linked gene 2-interacting protein X; H_2_O_2_, hydrogen peroxide.

Parent-cell engineering reshapes EVs composition at the source by preconditioning or genetically modifying MSCs to enrich specific therapeutic cargos (Kojima et al., 2018). For instance, hypoxic, cytokine, or pharmacological priming enhances the secretion of regenerative and antifibrotic factors, including miR-122, miR-181–5p, and anti-inflammatory cytokines such as IL-10, thereby improving inhibition of HSCs and promoting hepatocyte recovery (Yu et al., 2015; Zhang et al., 2020). Cargo engineering involves the active incorporation of defined RNA, protein, or small-molecule payloads into EVs using techniques such as electroporation, sonication, or membrane fusion. Rational loading of antifibrotic miRNAs (e.g., miR-29a and miR-122) or siRNAs targeting TGF-β/SMAD signaling components has been shown to enhance antifibrotic efficacy and regenerative signaling *in vivo* (Kamerkar et al., 2017; O'Brien et al., 2020; Wan et al., 2022). Surface engineering further improves delivery precision by decorating EVs membranes with targeting ligands through chemical conjugation or genetic fusion. Ligands such as RGD peptides, CXCR4, and N-acetylgalactosamine (GalNAc) confer enhanced liver tropism and facilitate selective interaction with activated HSCs and inflamed sinusoidal endothelium (D’Souza & Devarajan, 2015; Ferreira, Moreira and Rodrigues, 2022). Finally, biomaterial-assisted delivery systems—including injectable hydrogels, tissue scaffolds, and nanoparticle hybrids—enable controlled release, prolonged retention, and localized bioactivity of MSC-EVs within fibrotic tissues, thereby minimizing systemic loss and enhancing therapeutic durability (Kojima et al., 2018; Murphy et al., 2019). Collectively, these multidisciplinary engineering strategies converge to position MSC-EVs as clinically translatable, precision-engineered platforms for antifibrotic therapy.

### 4.1 Engineering at the parent MSCs level

MSCs have emerged as a preferred cellular platform for EVs production, owing to their minimally invasive sourcing, robust proliferative capacity, relatively juvenile epigenetic landscape, and immunological compatibility that supports allogeneic application (Sensebé and Bourin, 2009; Vizoso et al., 2017). Importantly, MSCs are highly amenable to upstream engineering, enabling precise control over EV yield, molecular composition, and functional potency. Such tunability is well aligned with the growing demands for product consistency, quality control, and reproducibility, as emphasized in the MISEV2023 guidelines for EV characterization and standardization (Welsh et al., 2024).

Genetic modification of MSCs constitutes a rational upstream engineering strategy to endogenously enrich EVs with therapeutic microRNAs (miRNAs), long non-coding RNAs (lncRNAs), or proteins, thereby enhancing cargo fidelity and functionally aligning EVs with defined antifibrotic pathways (Bellavia et al., 2017). For example, overexpression of miR-122, miR-181–5p, or miR-29a in MSCs enhances EV-mediated suppression of TGF-β/SMAD and PDGF/ERK signaling in HSCs, resulting in reduced collagen deposition and α-SMA expression (Lou et al., 2017b; Roderburg et al., 2011). Similarly, transcriptional engineering to upregulate HIF-1α or nuclear factor erythroid 2-related factor 2 (Nrf2) promotes antioxidant and pro-regenerative effects in hepatocytes by enriching EVs cargo with downstream effectors such as superoxide dismutase 2 (SOD2) and catalase (Bellezza et al., 2018). Compared with post-isolation cargo loading, cell-level engineering leverages endogenous EV biogenesis and sorting machinery, including nSMase2-, ALIX-, and Rab27a-mediated pathways, to achieve more consistent cargo enrichment, improved stability, and enhanced scalability (Ostrowski et al., 2010; Trajkovic et al., 2008). However, these approaches necessitate stringent biosafety control and regulatory evaluation, as stable genetic modification of parental cells is subject to increased scrutiny under advanced therapy medicinal product (ATMP) frameworks (Marks and Gottlieb, 2018).

Non-genetic priming provides a transient and tunable strategy to reprogram MSCs, thereby enhancing the functional potency of their secreted EVs. Hypoxic conditioning (1%–5% O_2_), for instance, activates HIF-1α-dependent transcriptional programs, resulting in EVs enriched with miR-21, vascular endothelial growth factor (VEGF), and angiopoietin-like 4 (ANGPTL4), which collectively promote hepatocyte survival and vascular remodeling (King, Michael and Gleadle, 2012). Similarly, cytokine priming using interferon-γ (IFN-γ) or tumor necrosis factor-α (TNF-α) enhances the immunomodulatory capacity of EVs by inducing the enrichment of factors such as indoleamine 2,3-dioxygenase (IDO), transforming growth factor-β3 (TGF-β3), and interleukin-10 (IL-10), thereby attenuating inflammatory activation of Kupffer cells and infiltrating macrophages (Françoiset al., 2012; Krampera et al., 2006). In addition, small-molecule priming with agents such as resveratrol, metformin, or 5-azacytidine can optimize mitochondrial function and modulate epigenetic states, generating EVs with enhanced antioxidant and pro-regenerative cargo profiles (Wang et al., 2016; Zhang et al., 2010). Notably, the efficacy of priming strategies is highly context-dependent, underscoring the need for standardized, omics-guided characterization and the definition of potency-linked critical quality attributes (CQAs) to support robust clinical translation (Lee et al., 2024; Takakura et al., 2024).

The transition from conventional two-dimensional (2D) monolayer culture systems to three-dimensional (3D) dynamic bioreactor platforms represents a critical advancement for scalable and good manufacturing practice (GMP)-compliant extracellular vesicle (EV) production. Three-dimensional culture configurations—including microcarrier-based stirred suspension bioreactors, vertical-wheel systems, and hydrogel-based scaffolds—have been shown to significantly enhance EVs yield (up to ∼20-fold) while preserving cellular phenotype and vesicle functionality (de Almeida Fuzeta et al., 2020; Haraszti et al., 2018; Patel et al., 2017). Notably, EVs derived from 3D-cultured MSCs exhibit increased microRNA diversity, improved hepatoprotective efficacy, and prolonged circulation half-life compared with those generated under conventional 2D conditions (Rocha et al., 2019). Furthermore, the integration of process analytical technologies (PAT) and closed, automated manufacturing systems enables real-time monitoring and control of critical process parameters, thereby ensuring batch consistency, sterility, and compliance with regulatory requirements for clinical-grade EV production (Lipsitz et al., 2016; Rathore et al., 2010). In parallel, multi-omics profiling and computational modeling are emerging as powerful tools to quantitatively link upstream bioprocess variables, including oxygen tension and shear stress, with downstream EV bioactivity, thereby enabling data-driven optimization and predictive control of EV manufacturing (Tsimberidou et al., 2020).

### 4.2 Cargo engineering of MSC-EVs

Cargo engineering aims to rationally modulate the molecular payload of EVs to enhance mechanism-specific therapeutic activity, potency, and reproducibility. In contrast to parent-cell or surface engineering, cargo engineering directly determines how EVs interact with intracellular signaling networks in HSCs, immune cells, and hepatocytes, thereby defining therapeutic specificity and efficacy. Endogenous cargo loading through genetic or epigenetic modification of MSCs enables selective and stable enrichment of regulatory nucleic acids or proteins into EVs via native vesicle biogenesis pathways. This upstream engineering strategy is particularly well-suited for liver fibrosis, a multifactorial disease requiring coordinated modulation of HSCs activation, macrophage polarization, and hepatocyte survival.

Mechanistically, MSCs engineered to overexpress antifibrotic microRNAs, including miR-122, miR-29a, and miR-181–5p, generate EVs that attenuate TGF-β/SMAD, PDGF/ERK, and YAP/TAZ signaling pathways in HSCs, thereby suppressing α-SMA expression and collagen deposition (Chen et al., 2023; Huang et al., 2017; Roderburg et al., 2011). In parallel, enrichment of anti-inflammatory cargos (e.g., miR-146a and miR-21) promotes macrophage reprogramming toward pro-resolving phenotypes, indirectly facilitating matrix degradation and hepatocyte regeneration (Hua et al., 2022). From a delivery and pharmacokinetic perspective, endogenous loading is highly compatible with systemic (e.g., intravenous) administration, as cargo-enriched EVs can exert therapeutic effects even with partial hepatic accumulation, provided sufficient biological potency is achieved. Moreover, as cargo incorporation is mediated by intrinsic sorting mechanisms involving ALIX, neutral sphingomyelinase 2 (nSMase2), and the ESCRT machinery, this approach enhances cargo stability and improves batch-to-batch consistency relative to post-isolation loading strate (Colombo, Raposo and Théry, 2014). Nevertheless, these advantages must be carefully balanced against regulatory considerations, as genetically modified MSCs master cell banks require rigorous safety assessment, including genomic stability, insertional mutagenesis risk, and long-term biosafety evaluation.

Exogenous cargo loading refers to the post-isolation incorporation of nucleic acids, proteins, or small-molecule therapeutics into MSC-EVs using physical or chemical techniques, including electroporation, sonication, membrane permeabilization, and lipid-based transfection. These approaches offer high modularity and rapid prototyping capacity, enabling flexible evaluation of diverse therapeutic cargos without permanent modification of parental cells (Kim et al., 2025; Lamichhane et al., 2016). However, exogenous loading strategies are frequently constrained by variable encapsulation efficiency, cargo aggregation, and partial disruption of EVs membrane integrity, which may in turn alter biodistribution profiles, cellular uptake pathways, and intracellular release kinetics (Kooijmans et al., 2013). In addition, inconsistencies in loading efficiency complicate dose-exposure-response modeling, thereby posing challenges for pharmacokinetic standardization and translational development. From a biomaterials perspective, comprehensive evaluation of exogenously loaded EVs should extend beyond nominal loading efficiency to include assessment of vesicle biophysical integrity, cargo retention and release kinetics, and mechanism-linked functional potency. Notably, recent studies integrating exogenous loading with biomaterial-assisted delivery platforms—such as injectable hydrogels and depot-forming matrices—have demonstrated enhanced cargo stability, prolonged retention, and sustained therapeutic exposure in fibrotic liver models (Kim and Lee, 2026; van de Looij et al., 2023; Yun et al., 2025).

Collectively, while exogenous cargo loading provides substantial engineering flexibility, its successful translation requires rigorous integration of materials characterization, pharmacokinetics, and mechanism-oriented bioactivity evaluation.

### 4.3 Surface engineering and targeting strategies for systemic delivery

Intravenous administration remains the most clinically practical route for MSC-derived EV–based therapies; however, systemic delivery is constrained by rapid clearance, nonproductive uptake by the MPS, and limited penetration into fibrotic niches. Consequently, surface engineering has emerged as a key strategy to enhance hepatic accumulation, cell-type-specific uptake, and overall bioavailability of EVs after systemic administration. From a biomaterials perspective, surface modification extends beyond simple biodistribution tuning to the deliberate reprogramming of nano-bio interactions at the interface between EVs, circulating proteins, and target cells, thereby improving therapeutic index while preserving vesicle integrity.

Ligand-mediated targeting strategies entail the display of affinity moieties, including peptides, carbohydrates, antibodies, or aptamers, on the EV membrane, thereby facilitating selective uptake by activated HSCs or liver-resident immune cells. Representative ligands include RGD peptides targeting integrins upregulated on activated HSCs, CXCR4-binding motifs exploiting chemokine gradients, and galactose or N-acetylgalactosamine (GalNAc) derivatives engaging the asialoglycoprotein receptor (ASGPR) on hepatocytes and sinusoidal cells (Kumar et al., 2023; Ruoslahti, 1996). Mechanistically, ligand-functionalized EVs exhibit enhanced receptor-mediated endocytosis, reduced nonspecific macrophage sequestration, and improved antifibrotic efficacy at lower administered doses compared with unmodified counterparts (Kumar et al., 2023). Importantly, ligand density and spatial presentation critically influence targeting efficiency, underscoring the need for quantitative surface characterization rather than qualitative confirmation alone. From an engineering standpoint, ligand-mediated targeting increases the effective local concentration at fibrotic sites, thereby compensating for incomplete hepatic accumulation and minimizing systemic exposure, which is an essential consideration for chronic antifibrotic therapy (Wang et al., 2026).

Beyond discrete ligand conjugation, membrane hybridization strategies generate chimeric vesicles through fusion of MSC-EVs with liposomes, synthetic nanoparticles, or cell-derived membranes. These hybrid systems integrate the biological functionality of EVs with the tunable physicochemical properties of engineered carriers, enabling prolonged circulation, enhanced cargo retention, and programmable surface composition (Sato et al., 2016; Zhang et al., 2019). In parallel, chemical modification approaches, including click chemistry, PEGylation, and covalent conjugation via amide or thiol linkages, enable precise control over ligand density and orientation on EV surfaces. While these strategies offer high reproducibility and modularity, they may introduce additional complexity, such as altered protein corona formation, potential immunogenicity, and challenges in large-scale manufacturing (Armstrong and Stevens, 2018; Kopac, 2021; Smyth et al., 2014). A recent study provides direct evidence that ligand engineering can reprogram the EV nano-bio interface for active targeting of fibrotic liver tissue. Human umbilical cord MSC-derived exosomes were functionalized with DSPE-PEG-aminoethyl anisamide (AEAA), which enabled recognition of sigma receptors enriched on activated hepatic stellate cells (Sun et al., 2025). Competitive ligand-blocking experiments reduced EV uptake, supporting a receptor-dependent targeting mechanism. When incorporated into a carboxymethyl chitosan/oxidized dextran/iron–tannate hydrogel, AEAA-modified exosomes exhibited sustained release, enhanced targeting of activated HSCs, and improved antifibrotic activity (Sun et al., 2025). This example illustrates that targeting is not determined by ligand identity alone but emerges from the combined effects of ligand density, orientation, spacer length, receptor availability, and the accessibility of the ligand after exposure to biological fluids and protein-corona formation.

From an engineering-oriented perspective, evaluation of surface-engineered MSC-EVs should extend beyond proof-of-concept targeting to encompass quantitative biodistribution profiling (e.g., whole-organ and cell-specific uptake), target-to-off-target uptake ratios under systemic conditions, correlation between enhanced targeting and functional antifibrotic efficacy, and assessment of immunogenicity, complement activation, and manufacturing scalability (Bertrand et al., 2014; Lee et al., 2018). Such multidimensional evaluation frameworks are essential to ensure that surface modification strategies translate into meaningful therapeutic benefit rather than incremental changes in biodistribution without functional gain. These processes are further shaped by dynamic nano–bio interface interactions that critically determine EV fate, tissue accessibility, and cellular uptake *in vivo*, as summarized in Figure 5.

**FIGURE 5:**
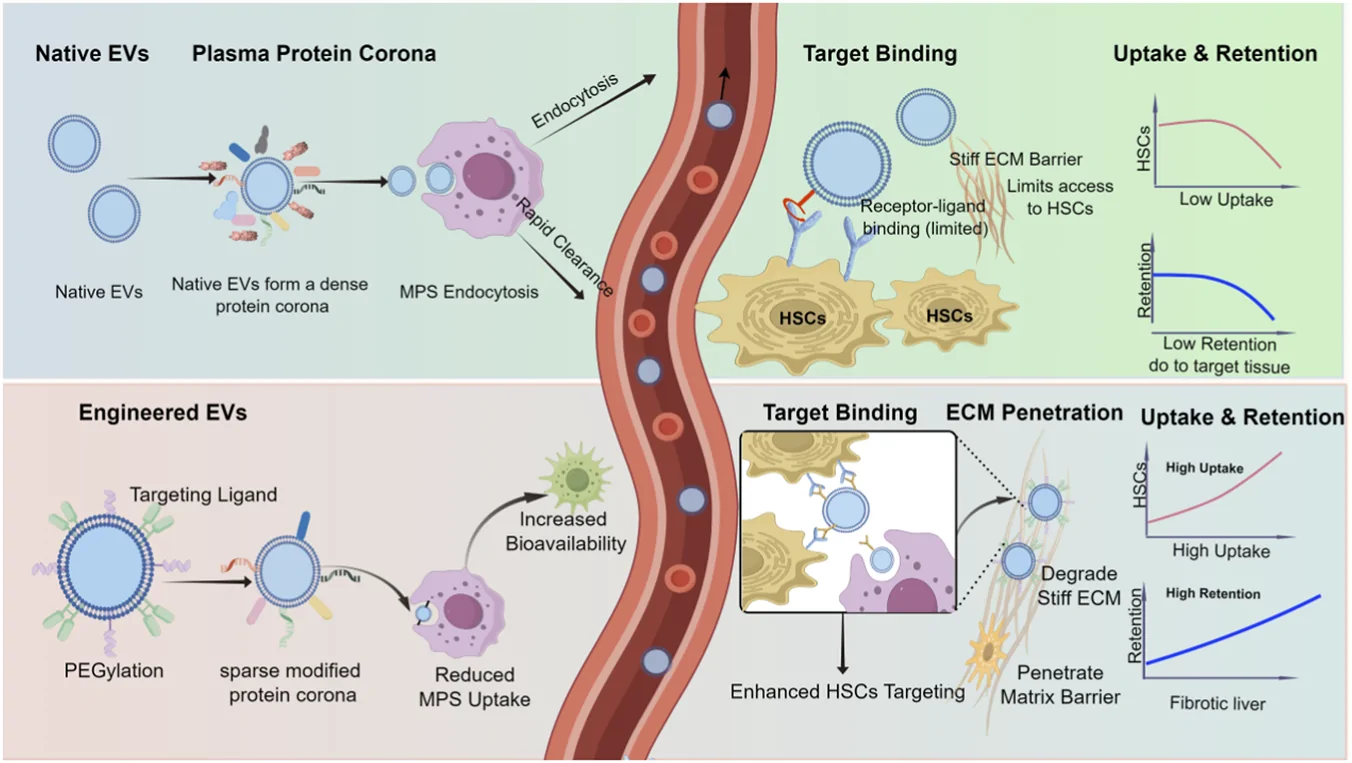
Nano–bio interface interactions governing the *in vivo* fate of engineered MSC-EVs in fibrotic liver. Following systemic administration, native MSC-EVs adsorb plasma proteins to form a dense protein corona, promoting MPS-mediated endocytosis and rapid clearance and thereby reducing circulating bioavailability. In fibrotic liver, the stiff ECM further restricts receptor–ligand interactions, tissue penetration, HSC uptake, and local retention. Surface PEGylation and ligand conjugation can limit nonspecific protein adsorption and MPS capture while enhancing receptor-mediated HSC targeting. Matrix-penetrating or matrix-remodeling modifications further facilitate transport across the fibrotic ECM, resulting in increased cellular uptake and tissue retention. Thus, coordinated regulation of protein-corona formation, immune recognition, target binding, and ECM penetration determines the *in vivo* fate and therapeutic performance of engineered MSC-EVs. Abbreviations: MSC-EVs, mesenchymal stem cell-derived extracellular vesicles; EVs, extracellular vesicles; MPS, mononuclear phagocyte system; PEG, poly (ethylene glycol); ECM, extracellular matrix; HSCs, hepatic stellate cells.

### 4.4 Biomaterial-assisted delivery and controlled release of MSC-EVs

To overcome the intrinsic limitations of bolus intravenous administration, biomaterial-assisted delivery systems are increasingly being explored as complementary and enabling strategies for MSC-EV-based therapy (Tibbitt and Anseth, 2009). Although systemic injection remains clinically practical, rapid clearance, transient exposure, and limited spatiotemporal control over EVs bioavailability constrain therapeutic durability, particularly in chronic diseases such as liver fibrosis. Injectable hydrogels, porous scaffolds, and hybrid nanocomposites can function as EVs depots, providing localized retention, sustained release, and physical protection against enzymatic degradation and premature clearance (Langer and Tirrell, 2004; Lu, Zhang and Ma, 2019). In preclinical models of liver fibrosis, such delivery platforms have been shown to prolong EV bioactivity, reduce dosing frequency, and enhance antifibrotic efficacy by maintaining therapeutically relevant concentrations within fibrotic niches over extended periods (Chen et al., 2013; Yu H. et al., 2025).

From a biomaterials perspective, the rational design of EV–matrix systems should consider key parameters, including material biocompatibility, degradation kinetics, EV–matrix interactions, and the preservation of vesicle integrity and bioactivity. Natural polymers, including alginate, gelatin, collagen, and hyaluronic acid, together with synthetic systems such as polyethylene glycol (PEG)-based hydrogels, provide tunable crosslinking densities and degradation profiles. However, the crosslinking mechanism itself can influence both EV quality and subsequent release behavior (Drury and Mooney, 2003; Gradinaru et al., 2018; Wang and Feng, 2023). Crosslinking chemistry should be considered a critical formulation attribute because it can affect EV membrane integrity and the accessibility of native surface proteins (Liu et al., 2025). Thermosensitive physical gelation generally avoids reactive crosslinkers and irradiation, but temperature shifts and hydrophobic polymer domains may alter membrane fluidity or promote vesicle aggregation. Photo-crosslinked GelMA offers tunable mechanical strength and retention; however, ultraviolet or visible-light exposure, photoinitiators, and radical intermediates may oxidize membrane lipids or surface proteins unless irradiation dose and initiator concentration are carefully controlled. Dynamic Schiff-base hydrogels form rapidly under aqueous and relatively mild conditions and provide injectability and self-healing, although residual aldehyde groups may react with primary amines on EV surface proteins and reduce the accessibility of tetraspanins or targeting ligands (Uman et al., 2024). Strain-promoted azide–alkyne or tetrazine-based bio-orthogonal reactions permit more selective immobilization under physiological conditions, whereas copper-catalyzed reactions or excessive surface derivatization may compromise vesicle function or impede release (Uman et al., 2024). Accordingly, EV size, aggregation, morphology, membrane integrity, cargo retention, uptake activity, and biological potency should be evaluated both before encapsulation and after release (Uman et al., 2024; Welsh et al., 2024). Surface accessibility of CD63, CD81, and engineered targeting ligands should be assessed separately because preservation of total protein abundance does not necessarily indicate preservation of exofacial epitopes (Welsh et al., 2024). Beyond its effects on EV integrity and bioactivity, the hydrogel network also reshapes the pharmacokinetic profile of delivered EVs. Biomaterial-assisted delivery enables decoupling of the total administered dose from peak systemic exposure, a critical consideration for safety and regulatory approval. By flattening pharmacokinetic profiles and avoiding high transient plasma concentrations, controlled-release systems may mitigate the risks of off-target immune activation, complement engagement, and cumulative toxicity, which are particularly relevant in the context of repeated or long-term antifibrotic therapy (Mardpour et al., 2019).

From an engineering and translational standpoint, these platforms further support multimodal design, enabling co-delivery of EVs with complementary therapeutic cues (e.g., anti-inflammatory agents or matrix-modulating factors) and compatibility with minimally invasive administration routes, such as intrahepatic or perivascular injection. Collectively, biomaterial-assisted delivery represents a powerful strategy to bridge the gap between EVs bioactivity and clinically meaningful therapeutic exposure. To bridge the gap between pathophysiological complexity and the selection of appropriate engineering strategies, we propose a rational design framework that systematically links disease-specific targets to corresponding engineering modules and desired functional outcomes, as illustrated in Figure 6.

**FIGURE 6:**
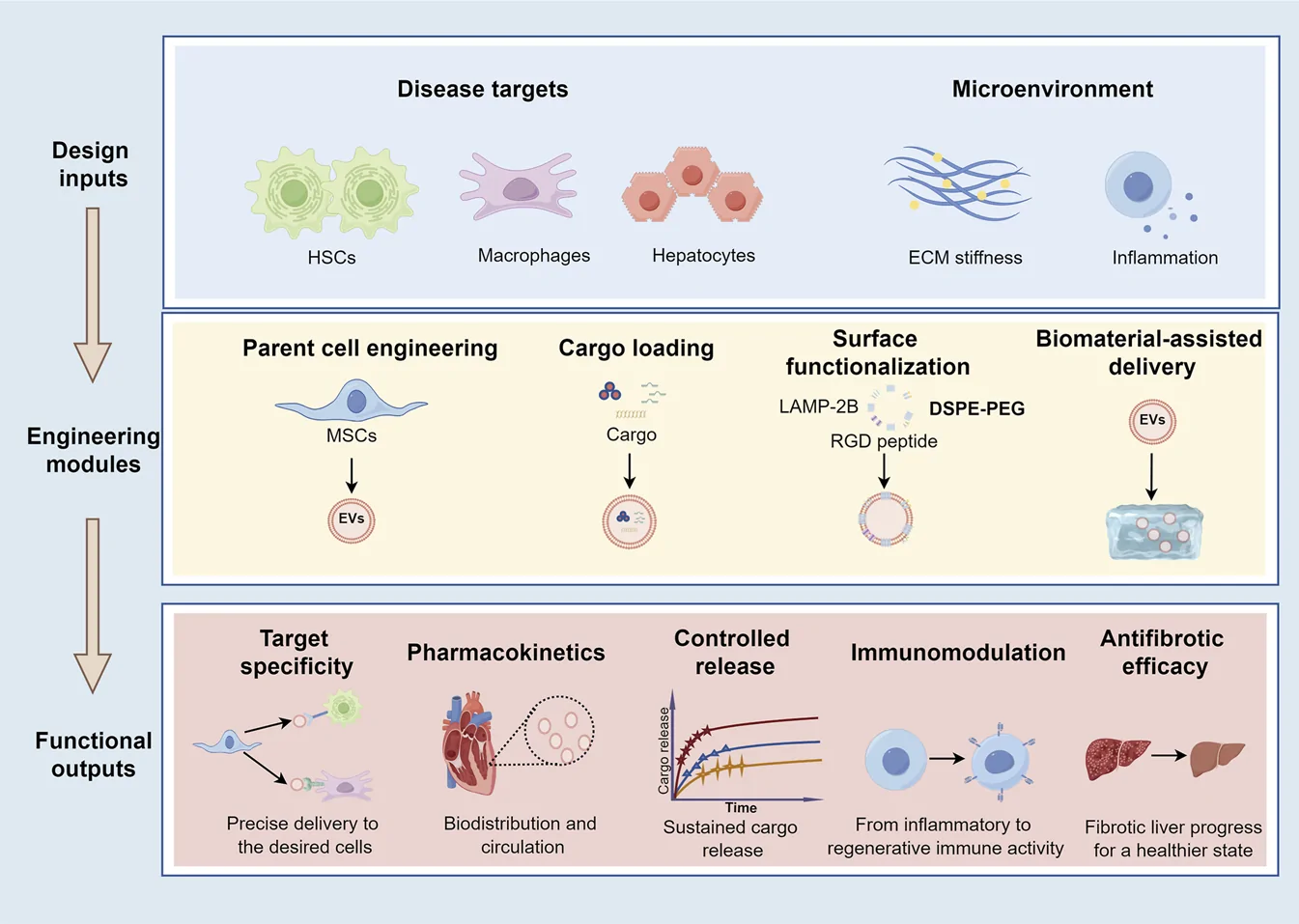
Design principles of engineered MSC-EVs as programmable biomaterial platforms for antifibrotic therapy. Disease-relevant cellular targets, including HSCs, macrophages, and hepatocytes, together with microenvironmental features such as ECM stiffness and inflammation, constitute the principal design inputs. These inputs guide four complementary engineering modules: parent-cell engineering, therapeutic cargo loading, surface functionalization using LAMP-2B, DSPE-PEG, or RGD peptides, and biomaterial-assisted delivery. Modular integration enables control over target specificity, pharmacokinetics and biodistribution, cargo-release kinetics, and immunomodulatory activity, thereby enhancing antifibrotic efficacy and promoting recovery toward a healthier liver state. Abbreviations: MSCs, mesenchymal stem cells; MSC-EVs, mesenchymal stem cell-derived extracellular vesicles; EVs, extracellular vesicles; HSCs, hepatic stellate cells; ECM, extracellular matrix; LAMP-2B, lysosome-associated membrane glycoprotein 2B; DSPE-PEG, 1,2-distearoyl-sn-glycero-3-phosphoethanolamine-poly (ethylene glycol); RGD, arginine-glycine-aspartic acid.

## 5 Manufacturing, quality control, and scalability of MSC-EVs

The successful clinical translation of engineered MSC-derived EVs depends on the alignment of therapeutic design with scalable manufacturing workflows. Key considerations include GMP-compatible production, standardized characterization, potency assays, stability assessment, and regulatory documentation. Integrating engineering-by-design with regulatory-by-design principles is essential to ensure reproducibility, batch consistency, and safety compliance. A translational roadmap linking production, quality attributes, and clinical deployment is presented in Figure 7. Consequently, engineering considerations must extend beyond biological efficacy to encompass integrated upstream bioprocessing, downstream purification, definition of critical quality attributes (CQAs), and product stability throughout the manufacturing lifecycle.

**FIGURE 7:**
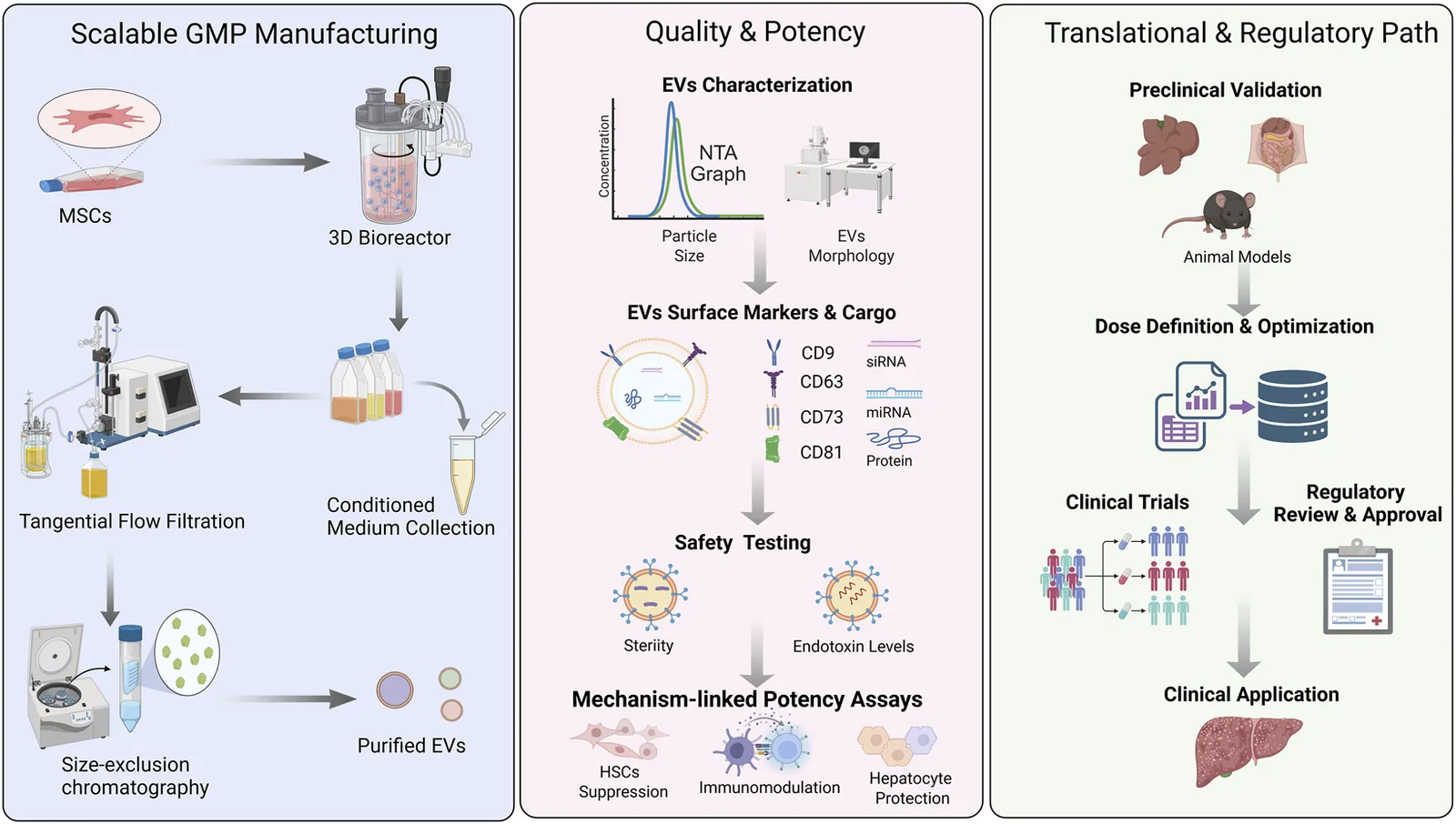
Scalable manufacturing, quality control, and translational roadmap of engineered MSC-EVs. GMP-compliant production begins with MSC expansion in three-dimensional bioreactors, followed by conditioned-medium collection, TFF, SEC, and recovery of purified EVs. Quality control encompasses particle concentration and size, morphology, surface-marker and cargo profiles, sterility, endotoxin levels, and mechanism-linked potency assays evaluating HSC suppression, immunomodulation, and hepatocyte protection. Translation proceeds through validation in relevant animal models, dose definition and optimization, clinical trials, regulatory review and approval, and eventual clinical application. This integrated framework links scalable manufacturing with predefined quality attributes, safety, biological potency, and regulatory requirements. Abbreviations: GMP, good manufacturing practice; MSCs, mesenchymal stem cells; MSC-EVs, mesenchymal stem cell-derived extracellular vesicles; EVs, extracellular vesicles; 3D, three-dimensional; TFF, tangential flow filtration; SEC, size-exclusion chromatography; NTA, nanoparticle tracking analysis; CD, cluster of differentiation; HSCs, hepatic stellate cells.

### 5.1 Alignment with MISEV2023 and implications for clinical translation

Within this manufacturing and quality-control framework, transparent reporting in accordance with community-endorsed standards is essential for evaluating the reproducibility and translational readiness of MSC-EV products. The reproducibility and translational interpretation of MSC-EV studies depend substantially on the extent to which EV production, separation, characterization, and functional testing are transparently reported (Théry et al., 2018; Van Deun et al., 2017). MISEV2023 recommends the use of operational EV terminology unless a specific biogenetic origin has been demonstrated, together with detailed reporting of MSC source, culture and conditioning parameters, separation and concentration procedures, particle abundance and size distribution, morphology, EV-associated components, and potential non-vesicular contaminants (Welsh et al., 2024). Across the representative studies reviewed, alignment with these recommendations remains heterogeneous, consistent with broader evidence demonstrating incomplete methodological reporting across the EV literature (Van Deun et al., 2017). Many studies combined differential ultracentrifugation or precipitation-based isolation with transmission electron microscopy, nanoparticle tracking analysis, and detection of canonical EV-associated proteins, such as CD9, CD63, CD81, TSG101, or ALIX. However, fewer studies reported negative or non-EV markers, co-isolated contaminants, separation recovery and specificity, normalization to the starting material, dose–response relationships, or potency-linked dose metrics. Moreover, the term “exosome” was sometimes used without direct evidence of endosomal biogenesis, for which the broader term “EV” or “small EV” would be more appropriate (Théry et al., 2018; Welsh et al., 2024). Engineered EV studies additionally require transparent reporting of the modification procedure, loading or conjugation efficiency, residual reagents, post-engineering identity and stability, and retention of biological activity (Welsh et al., 2024). Addressing these gaps through MISEV-aligned reporting, GMP-compatible manufacturing, validated identity, purity and potency assays, batch-consistency criteria, and stability testing will be essential for reproducible clinical translation. These requirements provide the methodological basis for the upstream production, downstream purification, quality-control, and formulation strategies discussed below.

### 5.2 Scalable upstream production of MSC-EVs

Mesenchymal stem cells are particularly well suited for large-scale EV manufacturing due to their robust proliferative capacity, efficient expansion under serum-free or xeno-free conditions, and relatively low donor-to-donor variability compared with other adult cell sources. These features support the establishment of standardized master cell banks and enable sustained, GMP-compliant production (Dominici et al., 2006; Galipeau and Sensébé, 2018). From a bioprocess engineering perspective, scalable EV production increasingly relies on 3D culture platforms, including microcarrier-based suspension systems, stirred-tank bioreactors, and vertical-wheel bioreactors. Compared with conventional 2D culture systems, 3D configurations enable precise control over oxygen tension, nutrient gradients, shear stress, and metabolic waste clearance, thereby enhancing EV yield, improving batch consistency, and increasing process robustness (Heathman et al., 2015; Mizukami and Swiech, 2018; Rathore and Winkle, 2009; Timmins and Nielsen, 2007).

Importantly, upstream process parameters exert a direct influence on downstream purification efficiency and final EV quality. Variations in culture conditions can affect EV size distribution, surface protein composition, and contaminant profiles. For example, excessive shear stress or suboptimal oxygenation may perturb EV biogenesis pathways and compromise vesicle integrity. Accordingly, integrated process design, supported by in-line monitoring and process analytical technologies (PAT), is essential to harmonize productivity with product quality and to enable reproducible, clinically compliant EV manufacturing (Mandenius and Brundin, 2008; Rathore and Winkle, 2009).

### 5.3 Downstream isolation and purification: from laboratory to GMP

While differential ultracentrifugation remains widely used in basic research, it is poorly suited for clinical translation due to low throughput, operator dependence, limited scalability, and the risk of vesicle aggregation or structural damage. In contrast, tangential flow filtration (TFF) combined with size-exclusion chromatography (SEC) has emerged as a more industry-compatible and good manufacturing practice (GMP)-aligned purification strategy for MSC-EVs (Busatto et al., 2018). TFF enables efficient concentration, diafiltration, and buffer exchange under closed-system conditions, whereas SEC further resolves EVs from soluble proteins, lipoproteins, and non-vesicular nanoparticles based on hydrodynamic size (Böing et al., 2014; Nordin et al., 2015). This modular purification workflow minimizes shear stress, preserves vesicle integrity, and supports linear scalability, rendering it particularly suitable for late-stage development and clinical manufacturing (Mol et al., 2017). From an engineering-oriented product perspective, purification processes should be optimized not only for particle yield but also for EVs purity, structural integrity, and preservation of biological function. Residual host cell proteins, nucleic acids, and process-related impurities must be quantitatively monitored and controlled as part of a critical quality attribute (CQA) framework (Giebel and Lim, 2025). Moreover, closed and automated purification systems support contamination control, batch traceability, and regulatory compliance, all of which are essential for scalable GMP manufacturing (Pedrioli et al., 2021). Collectively, the transition from laboratory-scale isolation to GMP-compliant purification represents a defining step in advancing MSC-EVs from promising biological entities to clinically viable therapeutic products.

### 5.4 Critical quality attributes and mechanism-linked potency assays

A central challenge in EVs therapeutics lies in defining CQAs that are predictive of *in vivo* efficacy. Standard physicochemical parameters, including particle size distribution, particle-to-protein ratio, canonical EVs marker expression, sterility, and endotoxin levels, are necessary but insufficient to guarantee consistent therapeutic performance (Reiner et al., 2017). Engineering-driven MSC-EVs development therefore requires mechanism-linked potency assays that reflect antifibrotic and immunomodulatory activity. Representative functional assays include: (i) suppression of HSCs activation markers (e.g., α-smooth muscle actin and collagen expression), (ii) modulation of macrophage inflammatory or pro-resolving phenotypes, and (iii) enhancement of hepatocyte survival and regenerative responses under stress conditions. Establishing quantitative relationships between CQAs and functional potency is essential for batch release, comparability assessment, and dose selection in clinical development.

### 5.5 Storage stability and formulation considerations

Long-term storage, transportation, and on-demand availability represent critical yet often underappreciated engineering challenges in the clinical translation MSC-EVs. The physicochemical stability of EVs is governed by multiple interrelated parameters, including storage temperature, buffer composition, ionic strength, freeze–thaw cycles, and formulation strategies, collectively determining vesicle integrity, cargo retention, and biological potency (Bosch et al., 2016; Jeyaram and Jay, 2017). Inadequate control of these variables may result in membrane disruption, vesicle aggregation, and degradation of bioactive components, ultimately compromising therapeutic efficacy and reproducibility.

Conventional cryopreservation at −80 °C remains the most widely adopted storage approach; however, repeated freeze-thaw cycles can induce membrane damage, promote vesicle aggregation, and lead to partial loss of RNA and protein cargo, thereby compromising functional activity (Lőrincz Á et al., 2014; Sokolova et al., 2011). Optimization of cryoprotective formulations, often involving stabilizing excipients such as trehalose, sucrose, or human serum albumin, can alleviate these effects by maintaining membrane integrity and minimizing ice crystal-induced damage, thereby emphasizing the need for formulation-specific stability assessment. Lyophilization, or freeze-drying, has emerged as a promising alternative by enabling room-temperature storage, facilitating transport, and reducing dependence on cold-chain infrastructure. However, this process can impose substantial physicochemical stress on EV membranes and their associated biomolecular cargo (Charoenviriyakul, Takahashi et al., 2018; Kusuma et al., 2018). Recent studies indicate that successful lyophilization requires careful selection of lyoprotectants, optimization of freezing and drying kinetics, and rigorous post-reconstitution validation to ensure preservation of particle size distribution, surface marker expression, and biological functionality (Kang et al., 2024; van der Meel et al., 2014). From a biomaterials perspective, lyophilized EV formulations should be regarded as complete drug products rather than intermediate materials, necessitating comprehensive evaluation of their quality, stability, and functional performance.

When integrated with biomaterial-assisted delivery systems, formulation complexity further increases. In EV-loaded hydrogels, porous scaffolds, or composite matrices, long-term stability should be evaluated not only at the level of individual vesicles but also with consideration of EV-matrix interactions, material degradation kinetics, and sustained release profiles. Mismatch between EV stability and matrix degradation profiles may result in premature loss of bioactivity or incomplete therapeutic release (Dec, Modrzejewski and Pawlik, 2022; Imai et al., 2015). Conversely, rational formulation design can enhance EV stability by shielding vesicles from enzymatic degradation, buffering pH fluctuations, and mitigating oxidative stress. Such strategies enable decoupling of manufacturing, storage, and administration timelines, which is particularly advantageous for off-the-shelf antifibrotic therapies requiring repeated or long-term dosing.

Collectively, rigorous evaluation of storage stability, formulation robustness, and post-storage functional potency is indispensable for the successful translation of MSC-EVs. From both engineering and regulatory perspectives, stability-indicating assays should be incorporated early in development pipelines to support shelf-life determination, comparability assessment, and eventual clinical deployment.

## 6 Translational considerations and clinical perspectives

Although substantial progress has been made in engineering MSC-EVs to improve cargo loading, targeting specificity, and sustained delivery, successful clinical translation ultimately depends on whether these design innovations can be translated into therapeutically actionable administration paradigms. In this context, the benefits conferred by EV engineering must be considered not only at the level of biological function, but also in relation to route of administration, dose intensity, exposure duration, biodistribution, and treatment schedule. These translational variables are particularly important in liver fibrosis, a chronic and spatially diffuse disease in which repeated systemic administration is likely to be required to achieve durable disease modification. Accordingly, clinical development of engineered MSC-EVs requires a pharmacologically informed framework that integrates delivery strategy with dosing design, safety considerations, and long-term treatment feasibility.

### 6.1 Route of administration and dosing strategies

Intravenous administration remains the most clinically feasible route for MSC-EVs therapy, particularly for diffuse liver diseases such as fibrosis, where localized delivery is often impractical. Systemic administration enables broad hepatic exposure through the portal and sinusoidal circulation; however, bolus intravenous injection is associated with rapid clearance, nonspecific sequestration by the MPS, and limited control over exposure duration (Takahashi et al., 2013). To address these limitations, engineering strategies—including EVs surface functionalization, ligand-mediated targeting, and biomaterial-assisted sustained-release systems—have been developed to enhance liver-specific accumulation and prolong therapeutic exposure (Tian et al., 2014). By reshaping pharmacokinetic profiles and reducing peak systemic concentrations, these approaches may improve therapeutic efficacy while mitigating safety risks associated with transient high-dose exposure.

Dose selection for EV-based therapeutics remains an unresolved and actively debated issue, as dosing metrics vary substantially across studies, including particle number, protein mass, and parent-cell equivalents (Pfaffl et al.,2025). Moreover, inconsistencies in EV characterization and potency further complicate cross-study comparisons and clinical translation. Importantly, engineering strategies that enhance intrinsic EV potency, cellular uptake efficiency, and tissue retention may enable lower effective doses and reduced dosing frequency, thereby expanding the therapeutic window and improving patient compliance (Antimisiaris et al., 2018; Bunggulawa et al., 2018). From a translational engineering perspective, rational dose design should integrate pharmacokinetic profiling, biodistribution analysis, and mechanism-linked potency assays, rather than relying solely on empirical scaling from preclinical models.

### 6.2 Safety and immunological considerations

MSC-EVs are generally regarded as low-immunogenic biological products, owing to their lack of replicative capacity, minimal expression of major histocompatibility complex (MHC) molecules, and intrinsic immunomodulatory properties (Tkach and Théry, 2016; Danger et al., 2016; Szebeni, 2005). In addition, advanced engineering strategies—such as surface-functionalized EVs, hybrid nanocarriers, and biomaterial-assisted delivery systems—introduce further complexity, including altered protein corona formation, material degradation byproducts, and prolonged or altered biodistribution profiles, all of which must be rigorously assessed for immunological compatibility and biodegradability (Monopoli et al., 2012).

From a translational engineering perspective, safety assessment should be integrated early into product design rather than deferred to late-stage validation. This includes *in vitro* hemocompatibility testing, complement activation assays, biodistribution and persistence studies, as well as repeat-dose toxicology models aligned with intended clinical use (Dobrovolskaia and McNeil, 2007; Park et al., 2008).

The incorporation of safety-by-design principles throughout the development pipeline not only reduces translational risk but also facilitates regulatory alignment and rational clinical trial design.

Collectively, the coordinated optimization of delivery routes, dosing strategies, and safety engineering will be essential to fully realize the therapeutic potential of MSC-EVs in liver fibrosis and related chronic diseases.

Although MSC-EVs avoid many risks associated with viable-cell transplantation, they are not biologically inert. Their safety is influenced by donor source, culture and preconditioning conditions, purification, storage, dose, administration route, and engineering strategy. Potential hazards include endotoxin or residual medium components, unintended bioactive cargo, excessive immunosuppression, and off-target tissue exposure (Takakura et al., 2024). Some MSC-EV preparations also express phosphatidylserine and tissue factor and exhibit procoagulant activity, which varies with cell source, culture conditions, and isolation method (Wright et al., 2023). Sterility, endotoxin, purity, tissue-factor activity, coagulation, complement activation, and cytokine release should therefore be incorporated into product characterization and batch-release criteria.

Surface engineering creates additional safety concerns by altering the biological identity of EVs beyond the intended targeting function. Lipid insertion, covalent conjugation, genetic surface display, and PEGylation may change particle size, surface charge, membrane fluidity, ligand density, and receptor interactions. Excessive modification may impair membrane integrity, reduce cellular uptake, mask native proteins, or expose novel epitopes. Accordingly, modification efficiency, free unconjugated ligands, residual reagents, aggregation, and the surface accessibility of targeting ligands and CD63/CD81 should be treated as critical quality attributes (Takakura et al., 2024).

The dynamic protein corona formed after systemic exposure is particularly important. Therapeutic EVs may acquire a primary corona during production and a secondary corona after contact with recipient plasma (Tóth et al., 2021). Immunoglobulins, complement components, fibrinogen, apolipoproteins, and other plasma proteins may mask targeting ligands or native tetraspanins, promote opsonization, and redirect engineered EVs toward Kupffer cells or splenic phagocytes (Tóth et al., 2021). However, corona formation is not uniformly detrimental: an albumin-enriched corona has been shown to reduce phagocytic uptake and enhance MSC-EV uptake by hepatocytes, liver sinusoidal endothelial cells, and hepatic stellate cells (Liam-Or et al., 2024). Targeting specificity should therefore be confirmed after incubation in disease-relevant human plasma rather than inferred solely from buffer-based binding and uptake assays.

PEGylation may reduce nonspecific protein adsorption and prolong initial circulation, but repeated administration can induce anti-PEG antibodies. In mice, PEGylated exosomes induced anti-PEG IgM and accelerated the clearance of subsequent doses, potentially reducing target-site exposure and altering biodistribution (Emam et al., 2021). Conjugated targeting peptides and proteins may likewise introduce immunogenic epitopes, particularly when they contain non-human sequences, form aggregates, or are displayed at high surface density. Nevertheless, direct repeat-dose immunogenicity data for ligand-decorated EVs remain limited; this should therefore be considered a product-specific translational risk rather than an established clinical outcome. Repeat-dose studies should assess anti-PEG and anti-ligand antibodies, post-serum ligand accessibility, receptor binding, complement activation, cytokine release, pharmacokinetics, and biodistribution (Emam et al., 2021; Takakura et al., 2024).

For EV-in-hydrogel formulations, safety evaluation should additionally consider residual crosslinkers or photoinitiators, degradation products, local inflammation, depot persistence, and unintended burst release (Liu et al., 2025). Although sustained-release systems may reduce peak systemic exposure, prolonged presentation of engineered EVs or foreign ligands may produce a different immune-exposure profile from bolus administration. A safety-by-design framework should therefore compare native EVs, engineered EVs, and released EVs using matched doses and include repeat-dose toxicology, hematological and coagulation analyses, organ histopathology, tissue persistence, immune responses, and cargo-specific off-target effects. Establishing the relationship among surface modification, corona evolution, immune recognition, biodistribution, and toxicity will be essential for defining a clinically acceptable therapeutic window.

### 6.3 Clinical development landscape

Early-phase clinical studies investigating MSC-EVs for liver diseases—including cirrhosis and acute-on-chronic liver failure—reflect a growing interest in cell-free regenerative and immunomodulatory therapies. To date, most completed and ongoing clinical investigations have focused on native, unmodified MSC-EVs rather than engineered variants; nevertheless, these studies provide valuable insights into clinical feasibility, preliminary safety, and dosing paradigms relevant to fibrotic liver diseases. Initial clinical evidence suggests that MSC-EVs administration is generally well tolerated, with no severe infusion-related adverse events reported in early-phase patient cohorts (Sengupta et al., 2020; Shi, Liu and Wang, 2011). However, substantial heterogeneity persists across studies in terms of dosing strategies, EVs characterization methods, and clinical endpoints, underscoring the urgent need for harmonization and standardization as the field advances. Although engineered MSC-EVs have not yet progressed to late-stage clinical trials in liver fibrosis, ongoing translational efforts in oncology, inflammatory disorders, and organ injury models demonstrate the technical feasibility of large-scale EVs manufacturing, quality control, and regulatory engagement (Lv et al., 2022). Importantly, insights gained from these indications, particularly with respect to repeat dosing, biodistribution, and long-term safety, are directly translatable to the setting of chronic liver fibrosis.

Looking forward, successful clinical translation will require the implementation of standardized and clinically meaningful endpoints. These include non-invasive fibrosis assessment methods (e.g., elastography-based liver stiffness measurement and circulating fibrosis biomarkers), functional liver parameters such as Model for End-Stage Liver Disease (MELD) score and Child-Pugh classification, as well as patient-reported outcomes reflecting quality of life (Archer et al., 2022). Given the chronic and progressive nature of liver fibrosis, long-term follow-up is critical for assessing the durability of therapeutic response, the extent of disease modification, and delayed adverse events, ultimately defining the true clinical value of MSC-EV-based therapies.

### 6.4 Regulatory and standardization challenges

Regulatory frameworks governing EV-based therapeutics are rapidly evolving and currently vary across jurisdictions. Unlike conventional biologics or cell-based therapies, EV-based products reside within a hybrid regulatory landscape, presenting challenges in product classification, potency definition, and comparability assessment (Tsuchiya et al., 2022). Harmonization of nomenclature, manufacturing standards, and reporting requirements is essential to enable cross-study comparability and support regulatory acceptance. International consensus initiatives, particularly the Minimal Information for Studies of Extracellular Vesicles (MISEV) guidelines, along with position statements from professional societies, have advanced the definition of minimal criteria for EV characterization, source attribution, and functional validation (Théry et al., 2018). Nonetheless, translating these scientific frameworks into regulatory-grade documentation, encompassing the definition of critical quality attributes (CQAs), the establishment of mechanism-linked potency assays, and the development of stability-indicating methods, remains a substantial challenge.

For engineered MSC-EVs, regulatory complexity is further amplified by upstream genetic modification, surface functionalization, and integration with biomaterial-based delivery systems. Each engineering layer introduces additional variables that must be justified in terms of risk-benefit balance, process control, and clinical necessity (Mendicino et al., 2014). Consequently, adopting a regulatory-by-design approach that aligns product design, bioprocess development, and quality management systems with regulatory expectations from the earliest stages is essential for streamlining clinical translation. Ultimately, successful clinical translation of MSC-EVs therapies for liver fibrosis will depend on the close integration of engineering innovation with regulatory science, supported by transparent reporting, harmonized standards, and early engagement with regulatory authorities. Such alignment will be critical to advance EV-based therapeutics from promising experimental platforms to approved and widely accessible clinical interventions.

## 7 Conclusions and future perspectives

Engineering has emerged as a defining and indispensable driver in the evolution of MSC-EVs from biologically active mediators into clinically credible therapeutic platforms for liver fibrosis. While early studies established the intrinsic anti-inflammatory and antifibrotic potential of MSC-EVs, it is now evident that translational success will depend not on biological activity alone, but on the depth and rigor of engineering integration (Vader et al., 2016). Through the coordinated implementation of parent-cell engineering, cargo and surface functionalization, biomaterial-assisted delivery, and scalable, GMP-compliant manufacturing, MSC-EVs systems can be transformed into precision therapeutics with defined mechanisms of action, improved targeting efficiency, and enhanced batch-to-batch consistency (Mayeen et al., 2025). Such integration facilitates controlled pharmacokinetics and alignment with regulatory expectations, thereby overcoming key barriers that have historically constrained the clinical advancement of EV-based therapies (Zuo et al., 2025).

Looking forward, continued progress will rely on the convergence of biomaterials science, bioengineering, and translational hepatology. In particular, the development of mechanism-linked potency assays that quantitatively link EV attributes to antifibrotic outcomes will be essential for rational dose selection and regulatory approval. In parallel, delivery system design should transition from empirically driven improvements in biodistribution to pharmacologically informed control of exposure–response relationships, especially in chronic diseases such as liver fibrosis (Tibbitt et al., 2016). Equally important is the early incorporation of regulatory-by-design principles, ensuring that manufacturing processes, quality documentation, and product specifications are aligned with emerging international standards for EV-based therapeutics. As regulatory frameworks continue to mature, platforms that integrate engineering precision, standardized characterization, and safety-by-design are most likely to progress from experimental constructs to clinically approved products (Théry et al., 2018).

In conclusion, engineered MSC-EVs represent a next-generation antifibrotic modality whose ultimate success will be determined not by biological promise alone, but by the extent of engineering integration across the entire product lifecycle. Sustained interdisciplinary collaboration will be essential to translate these versatile nanotherapeutics into safe, effective, and widely accessible treatments for liver fibrosis and related chronic liver diseases.

## Glossary

α-SMA: Alpha-smooth muscle actin
ATMP: Advanced therapy medicinal product
BDL: Bile duct ligation
**CCl**_**4**_: Carbon tetrachloride
CQA: Critical quality attribute(s)
DAMP: Damage-associated molecular pattern(s)
ECM: Extracellular matrix
ERK: Extracellular signal-regulated kinase
ESCRT: Endosomal sorting complex required for transport
EV: Extracellular vesicle(s)
GMP: Good manufacturing practice
HIF-1α: Hypoxia-inducible factor-1 alpha
HSC: Hepatic stellate cell(s)
IFN-γ: Interferon gamma
lncRNA: Long non-coding RNA
MCD: Methionine-choline-deficient (diet)
MELD: Model for end-stage liver disease
MISEV: Minimal information for studies of extracellular vesicles
MMP: Matrix metalloproteinase(s)
MPS: Mononuclear phagocyte system
MSC: Mesenchymal stromal/stem cell(s)
MSC-EV: Mesenchymal stromal cell-derived extracellular vesicle(s)
NF-κB: Nuclear factor kappa B
NK: Natural killer (cell)
Nrf2: Nuclear factor erythroid 2–related factor 2
PAT: Process analytical technology(ies)
PDGF: Platelet-derived growth factor
PDGFR: Platelet-derived growth factor receptor
PEG: Poly(ethylene glycol)
PI3K: Phosphoinositide 3-kinase
PK: Pharmacokinetics
QbD: Quality-by-design
ROCK: Rho-associated coiled-coil containing protein kinase
SEC: Size-exclusion chromatography
SMAD: Small mothers against decapentaplegic (proteins)
SOD2: Superoxide dismutase 2
TFF: Tangential flow filtration
TGF-β: Transforming growth factor beta
TIMP: Tissue inhibitor(s) of metalloproteinase(s)
TNF-α: Tumor necrosis factor alpha
UC-MSC: Umbilical cord-derived mesenchymal stromal/stem cell(s)
UC-MSC-EV: Umbilical cord MSC-derived extracellular vesicle(s)
VEGF: Vascular endothelial growth factor
